# Sensor for a Solid–Liquid Tribological System

**DOI:** 10.3390/s25020437

**Published:** 2025-01-13

**Authors:** Ruize Zhang, Zeyang Yu, Zhikai Fan, Shanshan Wang, Yihui Xiang, Yanfei Liu, Zhongnan Wang

**Affiliations:** 1 School of Mechanical Engineering, Beijing Institute of Technology, Beijing 100081, China; 3120220426@bit.edu.cn (R.Z.); 3120240437@bit.edu.cn (Z.Y.); 3120230406@bit.edu.cn (Z.F.); 2 Analysis & Testing Center, Beijing Institute of Technology, Beijing 100081, China; sswang@bit.edu.cn; 3 School of Chemistry and Chemical Engineering, Beijing Institute of Technology, Beijing 100081, China; 1120212912@bit.edu.cn; 4 School of Mechanical, Electronic and Control Engineering, Beijing Jiaotong University, Beijing 100044, China

**Keywords:** solid–liquid lubrication, sensors, acoustic, optical, coating

## Abstract

Solid–liquid lubrication systems have been widely used to enhance tribological behaviors. Alongside offering exceptional lubrication and wear-resistance performance, the active control of the tribological behavior of lubrication systems in accordance with service conditions is equally critical. To achieve this goal, accurately monitoring the condition of the lubrication system is fundamental. This review article aims to provide a fundamental understanding of different sensors for monitoring the condition of lubricants, as well as the friction and wear properties. Specifically, the sensors suitable for engineering applications are detailed introduced. Through this review, we wish to provide researchers in mechanical engineering with a clear technical overview, which can guide the design of intelligent lubrication systems with suitable sensors.

## 1. Introduction

Under the framework of sustainable human development, reducing the energy consumption of mechanical equipment has attracted increasing attention from both the research community and industry. Statistical analyses reveal that the phenomena of friction and the resultant wear are significant contributors to global energy expenditure and equipment malfunction rates. It is estimated that friction is responsible for the utilization of roughly one-third of the global primary energy resources. Additionally, wear-related issues lead to the failure of about 80% of mechanical components. Annually, the financial impact resulting from these dual factors is estimated to be between 2% and 7% of the respective countries’ gross domestic product [1].

Oil-based lubricants have been widely used to reduce friction and wear of moving components [2]. Various additives, including zinc dialkyl-dithiophosphate (ZDDP) [3,4,5,6], molybdenum dialkyl-dithiocarbamate (MoDTC) [7], and nanomaterials [8,9,10,11,12,13,14], have been applied to enhance lubrication and wear-resistance performance. Recently, with the rapid development of equipment, the operating conditions have become even harsher [15,16]. Taking electric vehicles (EV) as an example, the rotation speed of electrical motors for commercial EV cars has reached very high speeds (23,000 rpm for BYD; 27,200 rpm for XIAOMI V8s). High-performance lubricants with low viscosity are needed to reduce energy loss under high operating speeds. Against this background, eco-friendly water-based lubricants have been systemically investigated [17]. It has been found that superlubricity can be realized by water-based lubricants, which provides new possibilities for improving equipment performance [18]. Theoretically, superlubricity refers to a lubrication state where friction is almost or completely eliminated (with a friction coefficient below 0.01 and an extremely low wear rate) [19]. In-depth research on superlubricity is accompanied by the exploration of friction mechanisms, which not only reveal the essence of friction and lubrication but also hold significant value in the engineering field.

At the microscale, the Klein research group [20,21] found that the osmotic pressure between molecular brushes, the electrical double-layer forces, and the dispersion forces are key to achieving superlubricity. Subsequent studies have shown that surface charge [22] and surface ion adsorption [23] also significantly impact superlubricity behavior. At the macroscale, Tomizawa et al. [24] found that a silicon nitride friction pair lubricated with pure water can achieve superlubricity after a long period of running-in. The research group led by Prof. Luo achieved macroscopic superlubricity using phosphoric acid aqueous solution [25] and acids mixed with polyhydric alcohols [26,27] as lubricants. Recently, the research group led by Prof. Zhou designed and prepared hydrogels with multilayer structures that achieved macroscopic superlubricity over a wide range of loads [28]. Frictional chemical reactions, electrical double-layer forces, and hydration forces are considered the key microscopic interactive forces, as well as the synergistic effects of boundary films and fluid lubrication films, for achieving macroscopic liquid superlubricity. However, mono-liquid lubrication still has some inherent limitations, such as the wear of friction pairs with insufficient lubricant supply.

To address the shortcomings of mono-liquid lubrication systems, in recent years, researchers have conducted a series of studies on solid–liquid synergistic lubrication. Macroscopic superlubricity has been achieved using NbB_2_ thin films/pure water [29], DLC thin films/graphene oxide aqueous solution [30], graphene coatings/glycerol aqueous solution [31], and black phosphorus coatings/pure water [32], resulting in lower friction and wear compared to single solid or liquid lubrication materials. The synergistic effects of the boundary lubrication films formed by material adsorption [31], frictional chemical reactions [32], etc., and fluid effects are the main reasons for the realization of solid–liquid synergistic superlubricity.

In addition to providing excellent lubrication and wear-resistance performance, researchers also aim to control the tribological behavior of lubrication systems according to service conditions and the condition of liquid lubricants and solid lubricants. To achieve this goal, accurately monitoring the condition of the lubrication system is fundamental. The aim of this review article is to provide a fundamental understanding of different sensors for monitoring the condition of lubricants and the friction and wear properties. Coating-based sensors are also systemically introduced in this article. Through this review, we wish to provide researchers in mechanical engineering with a clear technical overview, which can guide the design of intelligent lubrication systems with suitable sensors.

## 2. Probing Lubricant Properties

Lubricating oil analysis is crucial for overseeing the wear, lubrication, and condition of oil-wetted moving pairs, offering early indications of potential mechanical component damage or failure. Over the years, both academic and industrial sectors have developed numerous monitoring techniques, primarily focused on measuring properties such as viscosity, total acid number (TAN), total base number (TBN), lubricant debris, water and aeration content, and elemental concentrations in lubricants.

Lubricant viscosity is a key parameter for assessing the condition of lubrication systems. Viscosity monitoring typically relies on sensor measurements of displacement, vibration, and acoustics. Displacement sensors, often adapted from laboratory methods like falling ball, rotary, and capillary viscometers, tend to be cumbersome and complex, with moving parts that can compromise reliability [33]. To address this, vibration and acoustic sensors have been developed for viscosity monitoring. One technique employs electromagnetically driven tuning forks to measure lubricant density and viscosity via frequency responses. The density and viscosity of lubricants are determined by recording the tuning forks’ frequency. This method can achieve accuracies of 0.01% for density and 1% for viscosity [34]. However, these devices remain relatively large for use as online sensors in lubrication oil monitoring systems. Among various sensors for vehicle lubrication systems, acoustic solid-state sensors, particularly Quartz Crystal Microbalance (QCM) sensors, have emerged as the most promising solution [33]. QCM sensors generate acoustic waves through alternating electrical fields, allowing viscosity to be monitored via the resonant frequency [35].

Lubricant condition assessment hinges on critical parameters like total acid number (TAN) and total base number (TBN). Typically, a rise in TAN signifies oxidation and acid contamination, whereas a decline in TBN indicates additive degradation. These changes can precipitate lubricant degradation or failure. Lubricant electrochemical behavior, influenced by acidity or basicity, can be monitored using various electrode-based techniques. For instance, Smiechowski and Lvovich [36] developed chronopotentiometric sensors using iridium oxide films to track these properties. The TAN and TBN were evaluated using the potential between iridium oxide electrodes. Additionally, ion-selective electrodes [37,38,39] have been employed to detect oil acidity changes, as electrode voltage negatively correlates with acidity. Beyond electrochemical methods, CO_2_ pressure sensors can monitor TBN, given that CO_2_ is a byproduct of the reaction between acid contaminants and CaCO_3_ [40]. A sensor that includes sputtered metals and glass molecularly bonded to a silicon substrate was used to evaluate the partial pressure of CO_2_. The rate of additive consumption in lubricants is mirrored by the partial pressure of CO_2_.

Water contamination in lubrication systems, often stemming from environmental condensation or coolant leakage, significantly contributes to wear debris formation and lubricant oxidation [41]. Schüller et al. [42] introduced a method to measure water concentration in oil/water dispersions, based on oscillator frequency changes influenced by the lubricant’s dielectric properties. However, the accuracy of this method is compromised by various factors affecting dielectric properties, such as water content, wear debris, and acidity [43]. Recent advancements include optical sensors [44,45] for rapid and straightforward water content monitoring. Optical fibers were used to measure the absorbance in the evanescent field in the spectral range of water absorption. Apart from water, air ingress, particularly in high-speed engines, can also exacerbate wear and oil oxidation. Although X-ray adsorption [46] and image analysis [47] methods can also monitor oil aeration rates, their stability and accessibility require further enhancement.

**Figure 1:**
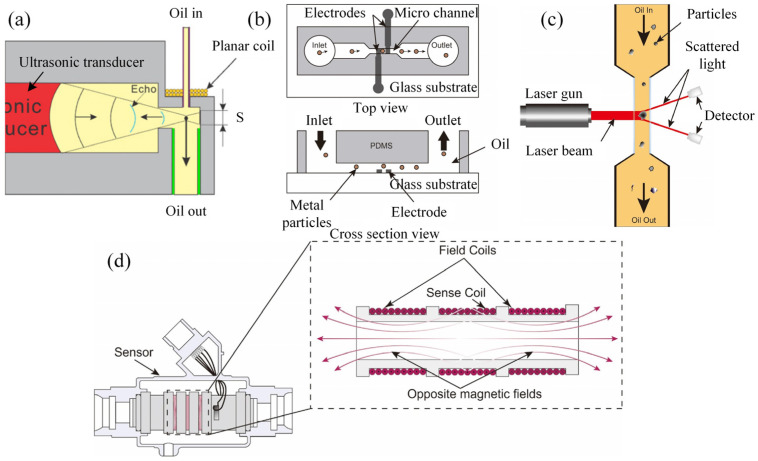
Schematic diagram of the wear debris monitoring techniques. (**a**) Acoustic-based ultrasonic-based oil debris sensor [50]; (**b**) electrical-based microfluidic capacitive Coulter sensor [53]; (**c**) optical-based light scattering wear debris sensor [60,61]; (**d**) magnetic-based Metalscan sensor [61,66].

Wear debris in lubricants, integral to assessing the tribological behavior of moving pairs, serves as a crucial indicator of machine wear and health through its concentration and particle size [48,49]. To facilitate real-time monitoring, a spectrum of techniques has been developed, categorized as acoustic, electrical, optical, and magnetic. Du and Zhe presented an ultrasonic pulse sensor (Figure 1a) [50], which guides the wear debris in lubrication oil to pass through the acoustic field. This process scatters the incident acoustic beam and generates an acoustic echo. The size of the debris is correlated with the acoustic echo, facilitating comprehensive counting and sizing of both metallic and non-metallic debris. Nemarich et al. [51] enhanced this with a three-transducer ultrasonic system to differentiate debris from air bubbles via comparative echo analysis, a method validated by Edmonds et al. [52] through distinct acoustic reflection coefficients. Despite their efficacy in distinguishing debris from bubbles, acoustic sensors struggle with identifying debris types due to similar reflection coefficients [50]. Electrical sensors, such as the microfluidic sensor by Murali et al. (Figure 1a) [53], employ the capacitance Coulter counting principle to size debris via pulse signal strength, though limitations in detecting dielectric debris and interference from water droplets hinder accuracy and application. Electrostatic sensors capitalize on the positive charge of adhesive wear debris [54,55,56,57]. Optical sensors, encompassing photoelectric and imaging types, leverage light extinction, scattering, and imaging technologies [58,59]. Light extinction and scattering sensors (Figure 1c) [60] effectively monitor debris over 5 μm, albeit with challenges from air bubbles, oil transparency, and debris overlap [61]. Imaging sensors, despite offering unique insights, face limitations from oil opacity and debris mobility, prompting research into online visual ferrographs for improved debris imaging [59,62,63], still constrained by image quality and debris overlap. Magnetic sensors, particularly magnetic chip detectors, are widely used in industry for capturing ferrous debris and generating pulse signals [64], yet they are limited to ferrous materials and lack single-particle information. Inductive sensors, responding to varying magnetic material properties, classify debris without throughput restrictions [65], but with limited debris type discrimination. Advances include triple-coil in-line inductive sensors (Figure 1d) [66] and microfluidic devices based on the inductive Coulter counting principle [67,68,69], offering high sensitivity, throughput, individual particle monitoring capacity, and accurate particle size detection.

The elemental composition of lubricant oil and wear debris serves as a proxy for lubricant condition and component wear. Spectroscopic methods are pivotal in this monitoring. Infrared (IR) spectroscopy, while informative on pollutants, additives, and oil quality, suffers from a limited elemental detection range and time-intensive data processing, thus constraining its practical use [70]. Fourier transform infrared (FTIR) spectroscopy, however, facilitates easier data handling, making it more apt for online monitoring [71,72]. X-ray fluorescence has been advanced to quantitatively analyze wear debris elements [73]. Despite the widespread application of these techniques in used oil analysis, real-time elemental monitoring remains challenging in industrial settings due to the complexity and cost of the equipment.

Currently, there is a significant push to prolong machine service life and maintenance intervals while minimizing lubricant use to mitigate environmental impact. To achieve this, multi-sensor systems are employed to gather comprehensive data on lubricant degradation. However, addressing the cross-sensitivity of these sensors requires efficient data processing solutions. Although online sensors for monitoring oil degradation are utilized in sectors like aviation and automotive, their application remains in the early stages. Future trends indicate a growing need for cost-effective, reliable, and compact sensors, which are essential for advancing the development and widespread adoption of oil condition monitoring technologies.

## 3. Probing Friction and Wear Properties

Monitoring the condition of lubricants is a widely used strategy to evaluate the condition of tribological systems. However, the performance of a lubrication system is hard to directly reflect through the lubricant condition. Friction and wear are the critical parameters to evaluate the performance of a lubrication system. In areas such as cutting and metal forming, both direct and indirect methods have been used to monitor the force during the friction process [74], including the usage of strain gauges [75] and displacement-based dynamometers [76], etc. However, for the moving parts in equipment like rolling and sliding bearings, it is difficult to monitor the friction force through such methods.

**Figure 2:**
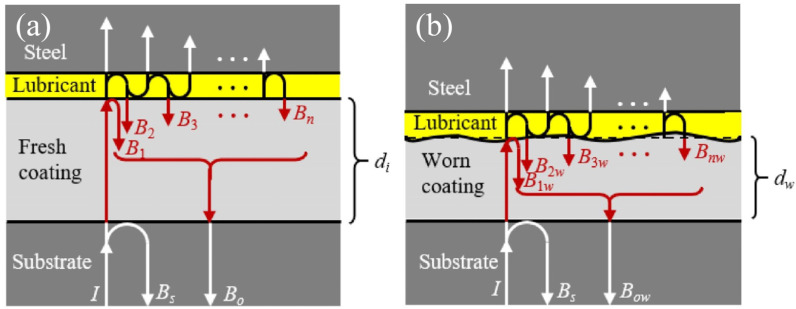
Schematic illustrating ultrasonic wave propagation through (**a**) an unworn coating–lubricant–steel assembly, and (**b**) a worn coating–lubricant–steel assembly with diminished coating thickness. Here, the worn coating thickness (*d*_w_) is defined as the initial coating thickness (*d*_i_) minus the wear depth, while d represents the lubricant film thickness [82].

Oil film thickness is important for determining the lubrication and wear behaviors, as it is highly related to the lubrication regime. Researchers have proposed various techniques to monitor the oil film thickness, with acoustic-based methods being the most widely used [77,78,79,80]. Wang et al. [81] introduced a multicycle focal spot mapping technique for determining the optimal pulse emission frequency and recorded pulse count. This method employs a series of ultrasonic pulses at a defined frequency to assess the lubricant film in roller bearings, ensuring that at least one pulse intersects the contact region’s center. The center oil film thickness is then derived from the minimum ultrasonic reflection coefficient amplitude. Statistical and experimental analyses confirm the method’s validity, demonstrating its capability to precisely measure the center oil film thickness in high-speed roller bearings, thereby surpassing the speed constraints of current techniques. Researchers have also proposed new techniques for the measurement of oil film thickness in solid–liquid lubrication systems incorporating surface coating. For sliding bearing, Dou et al. [82] presented an innovative ultrasonic reflection technique for concurrent measurement of the wear depth of the coating and film thickness of the lubricant (Figure 2), which can be determined by capturing the time or phase shift between reflected and incident waves, along with the amplitude reduction.

Moreover, Zheng et al. [83] introduced a pseudo-reflection coefficient, derived from the integrated reflected and reference waves, and developed a robust pseudo-reflection coefficient model for the concurrent determination of oil film and coating thickness. Acoustic simulations confirm the method’s accuracy and demonstrate superior stability of the pseudo reflection coefficient model.

**Figure 3:**
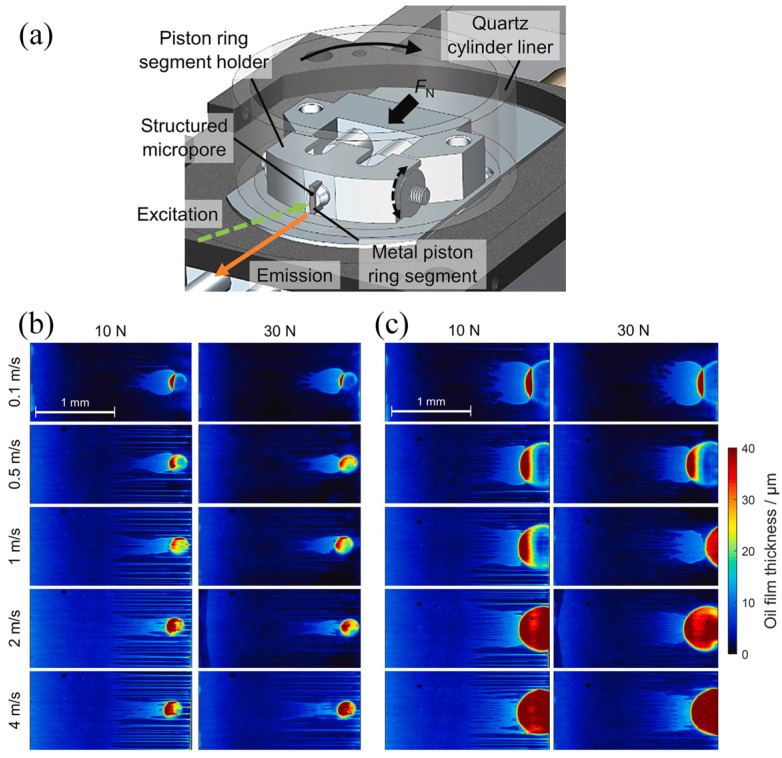
(**a**) Structure of the optical system. Oil tail images under sliding speeds ranging from 0.1 to 4 m/s and normal loads of 10 N and 30 N. (**b**,**c**) Images using surfaces with varied pore sizes of 200 μm and 500 μm, respectively [88].

Besides the acoustic-based techniques, optical-based techniques have also been used for oil film thickness measurement [84,85]. Zhang et al. [86] presented an experimental model utilizing ball-on-glass ring contact. An optical method is employed to analyze the steel ball–lubricant–chromium-coated glass ring system. Enhancements to the measurement system, based on optical analysis, facilitate the acquisition of high-quality interference images, enabling film thickness measurement under high-speed conditions. For the high-speed rolling bearing, the authors proposed a layered oil slip model incorporating slip and thermal effects, to investigate the film thickness at high operating speeds. The modified Reynolds equation, accounting for interfacial slip, is derived, with lubricant-limiting shear stress serving as the slip occurrence criterion. The model’s predicted film thickness closely aligns with experimental data, indicating that both interfacial slip and thermal effects significantly influence high-speed film thickness behavior. In addition, laser-induced fluorescence techniques have also been used to measure the oil film thickness [87,88]. Cheong et al. [88] investigated oil film behavior near a single surface texture in a sliding contact, mimicking the piston-ring/cylinder-liner interface in an engine. High-magnification laser-induced fluorescence was used for quantitative imaging of oil film thickness. It was revealed that the micropore transports oil to the starved outlet, forming a downstream “oil tail”. Importantly, at low speeds, the micropore generates a wider tail with a higher oil volume compared to high-speed conditions (Figure 3), indicating that liner porosity in reciprocating piston engines can enhance tribological performance.

**Figure 4:**
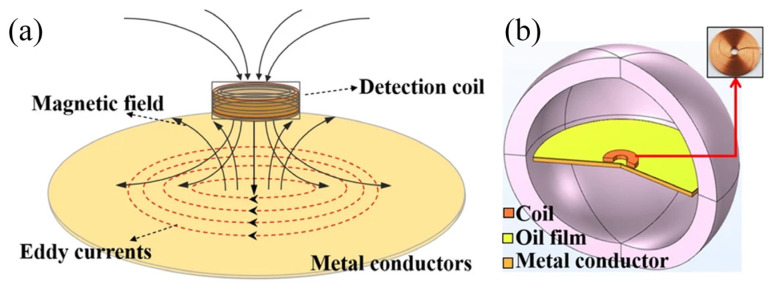
A model with an eddy current coil for oil film thickness detection is established by coupling electromagnetic and temperature fields. (**a**) Model for the proposed sensor, and (**b**) corresponding 3D model [89].

Optical methods can be used to accurately measure the film thickness of lubrication systems. However, the high demand for the transparency of contact materials largely suppresses their application for the online monitoring of lubrication conditions. Recently, Yuan et al. [89] proposed a high-precision method for simultaneously measuring lubricating oil film thickness and temperature using the eddy current effect (Figure 4). An oil film thickness detection model, incorporating an eddy current coil, was developed by coupling electromagnetic and temperature fields, elucidating the influence of temperature variations on film thickness measurement. A linear relationship was observed between coil inductance and film thickness, as well as between coil resistance and temperature, within specific ranges. A signal conversion module was designed, and a decoupling algorithm was proposed to extract thickness and temperature characteristics by separating the real and imaginary parts of the output voltage. Through comprehensive calculation and analysis, a fitting relationship was established between the voltage components and the oil film’s temperature and thickness, enhancing measurement accuracy and temperature stability, and enabling simultaneous sensing of both parameters.

## 4. Coating-Based Sensors

The malfunction of components within mechanical systems invariably results in diminished efficiency and subsequent economic setbacks. Illustratively, research has highlighted that 6.8% of the total downtime experienced by machining centers is attributable to the failure of machining tools [90]. To mitigate such downtime, it is imperative to monitor the damage and wear sustained by these components, a necessity that has spurred the evolution of wear sensors over time. Wear sensing methodologies can be broadly categorized into two types: direct measurement, which involves quantifying wear volume through optical techniques, and indirect measurement, which entails assessing wear based on monitored parameters such as acoustic emission sound, temperature, and vibration. However, direct monitoring methods often fall short in providing real-time wear data, necessitating the inference of wear conditions from parameters obtained through indirect monitoring. Furthermore, the real-time monitoring of critical parameters like load and temperature is equally paramount. In response to these challenges, coating sensors have been developed, designed to directly and continuously monitor the real-time condition of machine parts.

In the 1970s, the magnetic recording industry necessitated the development of coating sensors employing resistive technology, driven by the critical need for precise monitoring of the wear on recording heads, even in minute quantities [91]. These sensors typically comprise thin metal films deposited onto non-conductive substrates, wherein the resistance varies with the film’s wear, facilitating the assessment of wear through resistance monitoring. Nevertheless, temperature fluctuations can influence the monitored resistance, prompting the use of materials with lower temperature sensitivity to enhance the accuracy of resistive wear sensors, whereas materials with higher sensitivity are reserved for applications strictly involving thermal measurements [91].

Building upon the principles of coating wear sensors for magnetic recording heads and coating sensors for engine temperature measurement, Kreider and Ruff, in the 1990s, fabricated vacuum-deposited coating sensors to simultaneously monitor wear and temperature in bearing applications [92,93]. These laminated sensors, featuring one or more insulators or metallic films, were directly applied to the bearing surface, designed either to mimic the wear behavior of the bearing material or to be embedded as a small area within the bearing contour. The research identified adequate hardness, toughness, and low ductility as crucial for the conducting film, while low conductivity, hardness, and strength were essential for the insulating film. Additionally, high adhesion between the conducting and insulating films was paramount to ensuring the superior performance of the deposited coating sensors. These surface-deposited sensors are capable of continuously monitoring the wear depth and surface temperature of sliding bearings.

Sakka et al. [94] pioneered a wear sensing technique utilizing a sacrificial organic layer, wherein the temperature beneath this layer was monitored during friction tests to establish a correlation with wear depth. The parameters yielding the optimal correlation between wear depth and temperature were refined through numerical simulation. The study revealed that thermal conductivity is a critical factor in wear monitoring when employing an organic coating, with values below 1 W/m·K and above 4 W/m·K being particularly conducive to effective monitoring. Furthermore, the thickness of the coating was identified as having a significant impact on the performance of the wear monitoring system.

**Figure 5:**
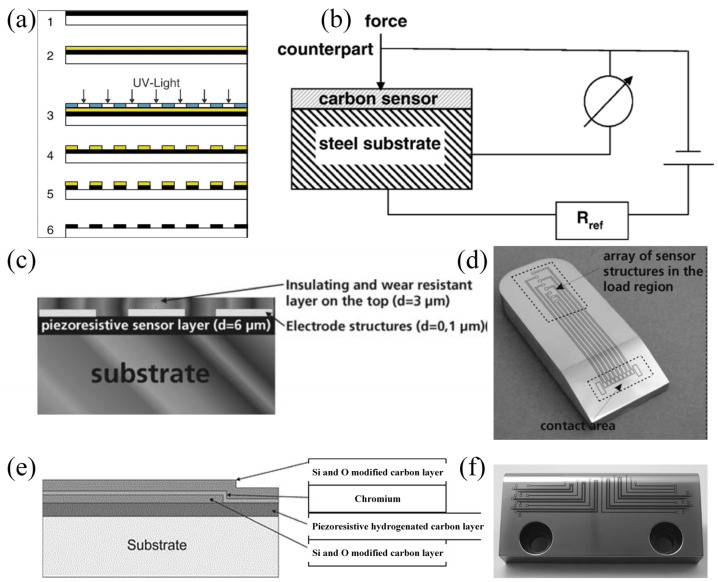
Development of the multi-functional DLC-based coating sensors. (**a**,**b**) Steps for the fabrication of electrodes for the sensor designed in 2006 and the corresponding schematic diagram of measurement [95]. (**c**,**d**) Schematic diagram of the modified coating sensor structure in 2010 and the corresponding picture of the fabricated coating sensor. An insulating and wear resistance layer was deposited on the top of the piezoresistive sensor layer as protection [98]. (**e**,**f**) The structure of the coating sensors was further modified into a multi-layer structure in 2016 for better performance [97].

Upon the deposition of coating sensors onto surfaces, the wear resistance of the sensor material emerges as a pivotal factor influencing the service life of these sensors. Amorphous diamond-like carbon (DLC), renowned for its superior lubrication and wear-resistance properties, has found extensive application in industrial contexts. Biehl et al. have demonstrated that DLC films exhibit pronounced piezoresistive behavior in addition to their exceptional tribological attributes, thereby rendering them suitable for direct load measurement on the friction surfaces of machine components (Figure 5a,b) [95]. Diverging from conventional strain gages employed as load sensors, coating sensors can be utilized in a rigid configuration, facilitating their application in both static and dynamic measurements. Subsequent advancements have led to the modification of the coating sensor system’s architecture (Figure 5c). A chromium (Cr) layer, serving as an electrode structure, has been deposited onto the piezoresistive DLC, followed by the deposition of an insulating and wear-resistant silicon-doped DLC layer atop for protective purposes. The sensor layer system exhibits minimal resistance upon losing contact with the workpiece, signifying a defect-free formation process. This characteristic endows the sensor with the capability to monitor both the load and the condition of the machining process, with potential applications extending to large technical components [96]. Further development of the coating sensor system has ensued [97], integrating a Cr meander for temperature monitoring and a DLC layer for load measurement into a unified coating sensor system (Figure 5e). This integrated system enables the simultaneous monitoring of two critical parameters, thereby unlocking promising application prospects across a myriad of industrial processes, such as strip drawing.

**Figure 6:**
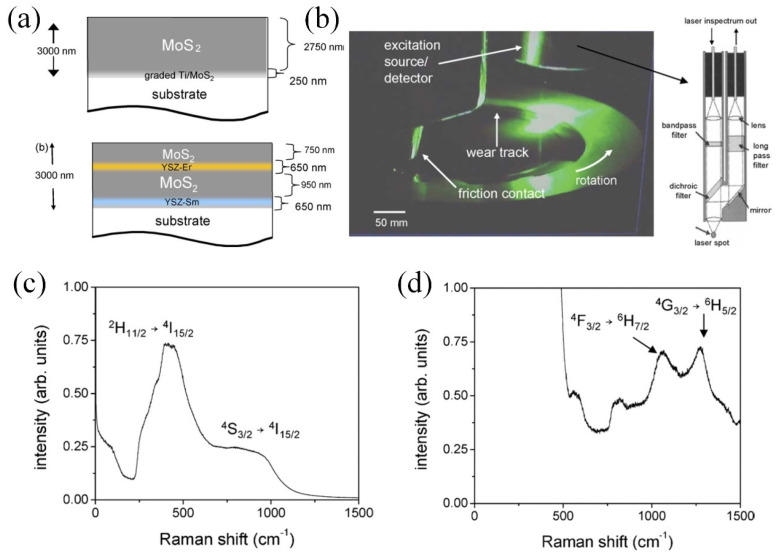
Wear sensor based on luminescence, where a luminescent coating is placed in the middle region of the multilayer coating for the detection of wear depth and warning of coating failure [101]. (**a**) Structure of the multilayer coating with embedded YSZ film as wear sensor. (**b**) Photograph of the monitoring system equipped on a tribometer, and the schematic of the laser probe. Luminescence spectra from (**c**) erbium-doped YSZ film and (**d**) samarium-doped YSZ film.

Significant advancements have been made in optical-based coating sensing technologies, notably through the creation of double or multilayer coatings, where each layer is characterized by unique optical properties to assess coating wear. Rasmussen et al. [99,100] pioneered a double-layer coating on a steel substrate, featuring a TiAlN layer overlying a TiN layer. Wear of the top TiAlN layer revealed the underlying TiN layer, which was easily detected by a simple optical imaging system due to a twofold increase in color values compared to the TiAlN layer, thereby streamlining wear monitoring of the coating system. Muratore et al. developed a MoS_2_ coating with embedded erbium-doped and samarium-doped yttria-stabilized zirconia (YSZ) coatings at the midpoint and the interface with the substrate, respectively, for luminescence-based health monitoring (Figure 6a) [101]. During friction, exposure of the YSZ layer in the multilayer coating to the surface allows activation by laser light (Figure 6b), emitting signals for wear monitoring. The mid-layer erbium-doped YSZ coating (Figure 6c) and the interfacial samarium-doped YSZ coating (Figure 6d) generate distinct signals, enabling straightforward determination of wear depth. Additionally, the embedded YSZ layer enhances the wear life of the single-layer MoS_2_ coating by over 30 times, due to the formation of a MoS_2_ transfer layer on the harder YSZ surface. Fang et al. introduced an Al/AlN multilayered coating system, incorporating an erbium-doped AlN layer as a luminescence sensor [102], showcasing the potential for wear sensing applications. Further research by Fang et al. on the impact of substrate temperature on the photoluminescence properties of the erbium-doped AlN film indicated that at a substrate temperature of 300 °C, the photoluminescence of Er^3+^ becomes visible and intense, thereby enhancing the suitability of the fabricated erbium-doped AlN film for wear monitoring applications [103].

**Figure 7:**
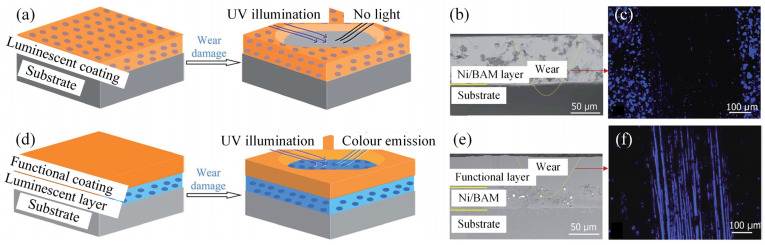
Different strategies for using luminescent layers in coating and their corresponding functional effects [106]. (**a**) Schematic diagrams of the coating with luminescent particles doped into the top layer. (**b**) Cross-sectional image of Ni/BAM layer deposited on the top of the substrate. (**c**) Luminescent image of the worn coating. (**d**) Schematic diagrams of the coating with luminescent particles doped into the mid-layer. (**e**) Cross-sectional image of coating with Ni/BAM layer between functional layer and substrate. (**f**) Luminescent image of the worn coating.

An alternative prevalent technique involves the incorporation of sensor materials with distinct optical properties into coatings. Salee et al. introduced luminescent epoxy resin coatings, embedded with ZnS:Cu powder [104] and silica/CdSe/ZnS quantum dots [105], beneath amorphous carbon layers for wear monitoring purposes. The residual thickness of the amorphous coating was accurately estimated by assessing the luminescent intensity, which corresponded well with measurements obtained using a profilometer [105]. He et al. [106] integrated BaMgAl_11_O_17_:Eu^2+^ (BAM) as a phosphor into Ni powder to create a Ni/BAM luminescent layer for coating health surveillance. This Ni/BAM layer could be positioned as the top layer (Figure 7a–c), where the disappearance of luminescence within the wear track region, contrasted by its presence in unworn areas, signified coating damage (Figure 7c). Alternatively, the Ni/BAM layer could serve as an intermediate layer between the top functional layer and the substrate (Figure 7d–f), with luminescence emerging upon the wear of the top functional coating (Figure 7f). Both configurations enabled effective monitoring of coating wear conditions. Furthermore, He et al. embedded sensor materials such as MAl_2_O_4_: Eu^2+^, Dy^3+^ (where M represents Sr or Ba), allowing for the identification of coating failure through the disappearance of luminescence under ultraviolet light [107]. Coating sensors, having been extensively employed to monitor the condition of mechanical components, are poised to drive the advancement of intelligent devices and industrial upgrading through the continued development of multi-functional coating sensors.

## 5. Self-Powered Sensors

**Figure 8:**
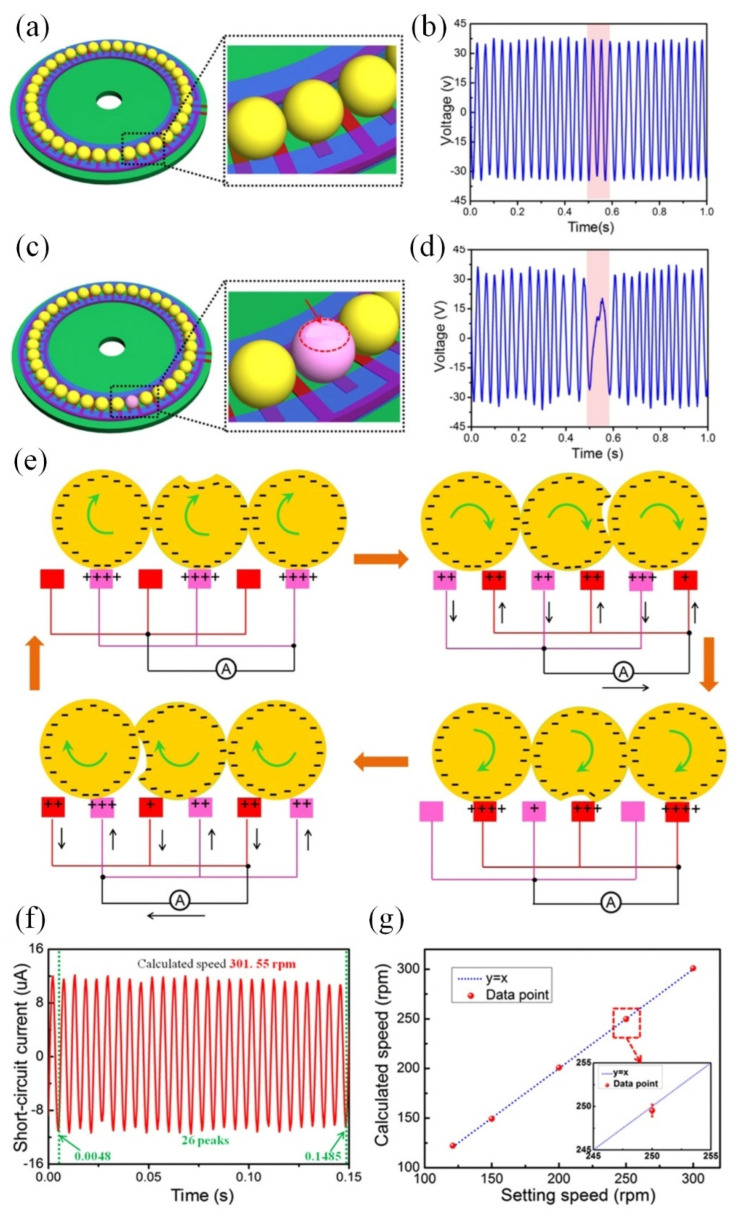
“Self-powered” sensor with bearing-based TENG [114]. (**a**) Schematic diagram of the unworn ball-bearing-based TENG sensor, where the red color corresponds to the interdigital electrode. (**b**) Output voltage with the unworn TENG sensor during the rotation process. (**c**) Schematic diagram of the ball-bearing-based TENG sensor with severe wear. (**d**) Output voltage with the worn TENG sensor during the rotation process. (**e**) Schematic diagrams of the working principles of the TENG with a severely worn bearing ball. (**f**) Calculation of rotation speed with the obtained signal. (**g**) Comparison between the calculated results and the set values. The calculated rotation speed is in accordance with the setting speeds.

Traditional coating sensors frequently require an external power source to continuously monitor sensor resistance or to operate light sources. However, the recent introduction of the “self-powered” sensor concept has ushered in a new era of maintenance-free and sustainable operation, holding significant potential for applications within wireless sensor networks, such as in robot systems [108] and condition monitoring systems [109]. Pioneered by Prof. Wang in 2012 [110], the Triboelectric Nanogenerator (TENG) stands out for its ability to convert mechanical energy into electrical energy via electrostatic induction and the triboelectric effect [111,112], thereby rendering it highly suitable for integration into “self-powered” sensor systems [113]. Li et al. [114] developed a TENG based on a ball-bearing structure, featuring Cu interdigital electrodes deposited on a glass epoxy substrate (Figure 8). The sensor’s output is derived from the rolling electrification that occurs between the bearing balls and the interdigital electrodes, enabling the monitoring of bearing ball wear through fluctuations in output voltage during rotation (Figure 8a–e). Additionally, the rotation speeds can be readily determined by analyzing the periodic output signals (Figure 8f,g).

**Figure 9:**
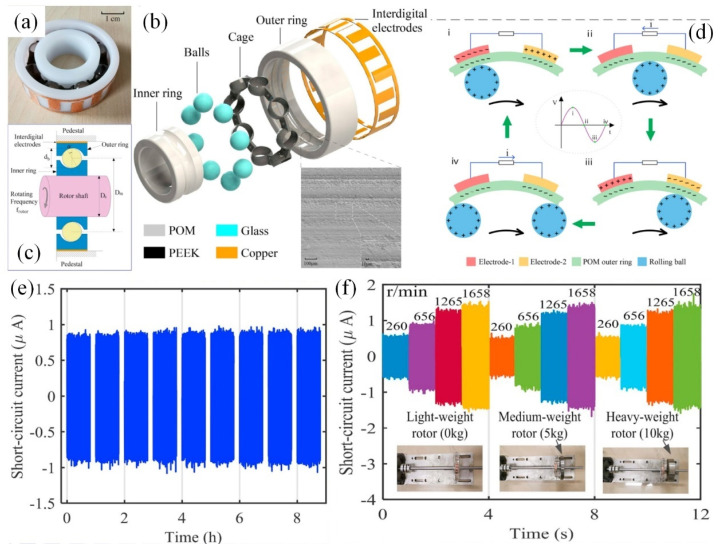
TRBB with self-powering and self-sensing performances [115]. (**a**) Photograph, (**b**) schematic diagram, and (**c**) cross-sectional structure of the TRBB, where an interdigital electrode was placed on the surface of the outer ring of the bearing to avoid direct contact between the bearing balls and the electrode. (**d**) Schematic working principle of the TRBB at different free-standing modes during the rolling process. (**e**) Durability test results indicate that the TRBB can provide stable short-circuit current. (**f**) Test results indicate that the TRBB can successfully monitor rotation speeds under different loads.

Leveraging the principles of Triboelectric Nanogenerators (TENGs), Han et al. [115] innovated a triboelectric rolling ball bearing (TRBB) that demonstrated both self-powering and self-sensing capabilities (Figure 9). The design incorporated an interdigital electrode on the outer ring’s surface, strategically positioned to preclude direct interaction between the bearing balls and the electrode while preserving the self-powering functionality (Figure 9d). This innovative design significantly enhances the service life of the TRBB (Figure 9e), and the correlation between changed frequency, output current, and rotation speeds facilitates its application in monitoring rotational velocities. Recently, for solid–liquid systems, Suh et al. [116] explored the effects of different solid materials, including both conductors and insulators, and assessed the impact of the liquid triboelectric series. The optimization of the contact electrification was summarized for the further improvement of TENG.

## 6. Conclusions

Solid–liquid lubrication systems are extensively employed to augment tribological performance. Recent studies suggest that excellent performance can be achieved by solid–liquid tribological systems [117,118,119]. To achieve precise control over the tribological behavior of these systems and to ascertain their service duration, it is imperative to utilize advanced sensor technologies for accurate condition monitoring. Researchers have proposed various technologies, and these sensors have found widespread application in engineering scenarios. For more intricate solid–liquid lubrication systems, precise characterization of the properties of individual solid and liquid materials is essential, and the integration of advanced active lubrication control technologies is recommended.

Nowadays, sensors for oil condition monitoring are widely used in the automotive industry. Moreover, acoustic-based oil film sensors are already being employed to monitor lubrication conditions. In the future, advanced tribological sensors can be combined with friction-active control technologies to realize intelligent lubrication systems, which would offer better adaptivity for complicated service conditions.

## Data Availability

Data available on request.
